# Alterations in ALK/ROS1/NTRK/MET drive a group of infantile hemispheric gliomas

**DOI:** 10.1038/s41467-019-12187-5

**Published:** 2019-09-25

**Authors:** Ana S. Guerreiro Stucklin, Scott Ryall, Kohei Fukuoka, Michal Zapotocky, Alvaro Lassaletta, Christopher Li, Taylor Bridge, Byungjin Kim, Anthony Arnoldo, Paul E. Kowalski, Yvonne Zhong, Monique Johnson, Claire Li, Arun K. Ramani, Robert Siddaway, Liana Figueiredo Nobre, Pasqualino de Antonellis, Christopher Dunham, Sylvia Cheng, Daniel R. Boué, Jonathan L. Finlay, Scott L. Coven, Inmaculada de Prada, Marta Perez-Somarriba, Claudia C. Faria, Michael A. Grotzer, Elisabeth Rushing, David Sumerauer, Josef Zamecnik, Lenka Krskova, Miguel Garcia Ariza, Ofelia Cruz, Andres Morales La Madrid, Palma Solano, Keita Terashima, Yoshiko Nakano, Koichi Ichimura, Motoo Nagane, Hiroaki Sakamoto, Maria Joao Gil-da-Costa, Roberto Silva, Donna L. Johnston, Jean Michaud, Bev Wilson, Frank K. H. van Landeghem, Angelica Oviedo, P. Daniel McNeely, Bruce Crooks, Iris Fried, Nataliya Zhukova, Jordan R. Hansford, Amulya Nageswararao, Livia Garzia, Mary Shago, Michael Brudno, Meredith S. Irwin, Ute Bartels, Vijay Ramaswamy, Eric Bouffet, Michael D. Taylor, Uri Tabori, Cynthia Hawkins

**Affiliations:** 1 Developmental and Stem Cell Biology Program, The Hospital for Sick Children, Toronto, ON Canada; 2 The Arthur and Sonia Labatt Brain Tumor Research Centre, The Hospital for Sick Children, Toronto, ON Canada; 3 Department of Hematology and Oncology, The Hospital for Sick Children, Toronto, ON Canada; 4 Department of Oncology and Children’s Research Center, University Children’s Hospital Zurich, Zurich, Switzerland; 5 Department of Laboratory Medicine and Pathobiology, University of Toronto, Toronto, ON Canada; 6 Second Faculty of Medicine, Charles University and University Hospital Motol, Prague, Czech Republic; 7 Department of Pediatric Hematology and Oncology, Hospital Universitario Niño Jesús, Madrid, Spain; 8 Department of Pediatric Laboratory Medicine, The Hospital for Sick Children, Toronto, ON Canada; 9 Centre for Computational Medicine, The Hospital for Sick Children, Toronto, ON Canada; 10 Division of Anatomic Pathology, British Columbia Children’s Hospital, Vancouver, BC Canada; 11 Department of Pathology and Laboratory Medicine, The University of British Columbia, Vancouver, BC Canada; 12 Department of Pediatrics, The University of British Columbia, Vancouver, BC Canada; 13 Division of Hematology/Oncology/BMT, British Columbia Children’s Hospital, Vancouver, BC Canada; 14 Department of Pathology and Laboratory Medicine, Nationwide Children’s Hospital, Columbus, OH USA; 15 Department of Pathology, The Ohio State University College of Medicine, Columbus, OH USA; 16 Division of Hematology/Oncology/Bone Marrow Transplantation, Nationwide Children’s Hospital, Columbus, OH USA; 17 Department of Pathology, Hospital Universitario Niño Jesús, Madrid, Spain; 18 Division of Neurosurgery, Centro Hospitalar Lisboa Norte, Hospital de Santa Maria, Lisbon, Portugal; 19 Instituto de Medicina Molecular João Lobo Antunes, Faculdade de Medicina, Universidade de Lisboa, Lisbon, Portugal; 20 Institute of Neuropathology, University Hospital Zurich, Zurich, Switzerland; 21 Department of Pediatric Oncology, Hospital Cruces, Bilbao, Spain; 22 Department of Pediatric Oncology, Hospital Sant Joan de Déu, Barcelona, Spain; 23 Department of Pediatric Oncology, Hospital Infantil Virgen del Rocio, Sevilla, Spain; 24 Children’s Cancer Center, National Center for Child Health and Development, Tokyo, Japan; 25 Division of Brain Tumor Translational Research, National Cancer Center Research Institute, Tokyo, Japan; 26 Department of Neurosurgery, Kyorin University Faculty of Medicine, Tokyo, Japan; 27 Department of Pediatric Neurosurgery, Osaka City General Hospital, Osaka, Japan; 28 Division of Pediatric Hematoncology, University Hospital de São João, Porto, Portugal; 29 Department of Pathology, University Hospital de São João, Porto, Portugal; 30 Division of Hematology/Oncology, Children’s Hospital of Eastern Ontario, Ottawa, ON Canada; 31 Department of Pathology and Laboratory Medicine, University of Ottawa, Ottawa, ON Canada; 32 Department of Pediatrics, University of Alberta, Edmonton, AB Canada; 33 Department of Laboratory Medicine & Pathology, University of Alberta, Edmonton, AB Canada; 34 Department of Anatomic Pathology, Dalhousie University, Halifax, NS Canada; 35 Department of Pathology Laboratory Medicine, IWK Health Centre, Halifax, NS Canada; 36 Division of Neurosurgery, IWK Health Centre, Halifax, NS Canada; 37 Division of Hematology-Oncology, IWK Health Centre, Halifax, NS Canada; 38 The Department of Pediatric Hematology Oncology, Hadassah Medical Center, Jerusalem, Israel; 39 Children’s Cancer Centre, Royal Children’s Hospital, Melbourne, Australia; 40 Murdoch Children’s Research Institute, Department of Paediatrics, University of Melbourne, Melbourne, Australia; 41 Division of Pediatric Hematology/Oncology, Mayo Clinic, Rochester, MN USA; 42 Department of Medicine, McGill University, Montreal, QC Canada; 43 Department of Neurosurgery, The Hospital for Sick Children, Toronto, ON Canada; 44 Department of Medical Biophysics, University of Toronto, Toronto, ON Canada

**Keywords:** Cancer genomics, Molecular medicine

## Abstract

Infant gliomas have paradoxical clinical behavior compared to those in children and adults: low-grade tumors have a higher mortality rate, while high-grade tumors have a better outcome. However, we have little understanding of their biology and therefore cannot explain this behavior nor what constitutes optimal clinical management. Here we report a comprehensive genetic analysis of an international cohort of clinically annotated infant gliomas, revealing 3 clinical subgroups. Group 1 tumors arise in the cerebral hemispheres and harbor alterations in the receptor tyrosine kinases *ALK*, *ROS1*, *NTRK* and *MET*. These are typically single-events and confer an intermediate outcome. Groups 2 and 3 gliomas harbor *RAS/MAPK* pathway mutations and arise in the hemispheres and midline, respectively. Group 2 tumors have excellent long-term survival, while group 3 tumors progress rapidly and do not respond well to chemoradiation. We conclude that infant gliomas comprise 3 subgroups, justifying the need for specialized therapeutic strategies.

## Abstract (web summary)

Infant gliomas behave differently to their childhood or adult counterparts. Here, the authors perform a large-scale genetic analysis of these tumours, revealing genetic alterations which may offer therapeutic opportunities.

## Introduction

Gliomas are the most common primary central nervous system (CNS) neoplasm and result in the highest tumor-associated morbidity and mortality in children and adults^1,2^. Traditionally, gliomas are divided into low grade (LGG, WHO grades I–II) and high grade (HGG, WHO grades III–IV) based on their histological characteristics^3^. Molecularly, adult lower grade gliomas commonly harbor alterations in *IDH1/2* in association with *TP53* and *ATRX* mutations or *TERT* mutations and 1p/19q co-deletions^4^. In comparison, most childhood LGG are driven by *RAS/MAPK* activation—predominantly in the form of fusions or mutations involving the *BRAF* gene—and rarely undergo malignant transformation^5–7^. In contrast, adult LGG rarely contain *RAS/MAPK* alterations^8^ and invariably transform to HGG over time^9^. Pediatric HGG are usually not the result of transformation from LGG and, in contrast to adult HGG, most commonly harbor recurrent mutations in the genes encoding histone H3.3 and H3.1^10,11^.

In contrast to the abundance of genetic and clinical information now available for pediatric glioma, far less is known about the infant demographic (under 1 year of age), despite the incidence of CNS tumors being highest in this group^1^. Although steady improvements in the overall outcome of childhood cancer have been observed globally, infants with brain tumors remain at high risk for early death after diagnosis, are less likely to be enrolled in clinical trials and are critically under-studied^12^. Further, the association between tumor grade and outcome is less predictable in infants; infant LGG (iLGG) show a more aggressive course^13–15^, while infant HGG (iHGG) have a better outcome^16,17^ when compared with older children and adolescents. As such, the classic treatment approaches for pediatric LGG (low dose chemotherapy) and HGG (surgery, radiation and alkylator-based chemotherapy) are often either ineffective or excessive, respectively. Therefore, clinicians caring for infants with gliomas are faced with the challenging task of treating an exceptionally vulnerable population of patients where the best treatment options remain ambiguous and data are scarce.

To address the lack of knowledge regarding the genetic underpinnings of infant gliomas, we assemble a multi-institutional, international collaborative taskforce to comprehensively characterize a large, clinically well-annotated cohort with follow-up data spanning three decades. We find that infant gliomas comprise three main subgroups: (1) hemispheric receptor tyrosine kinase (RTK)-driven tumors, including *ALK*, *ROS1, NTRK,* and *MET* fusions, which are enriched for HGG and have an intermediate clinical outcome, (2) hemispheric *RAS/MAPK*-driven tumors, which show excellent long-term survival with minimal clinical intervention post-surgery, and (3) midline *RAS/MAPK*-driven tumors, which are enriched for LGG with *BRAF* alterations and have a relatively poor outcome even after conventional chemotherapeutic approaches. Together the clinical and molecular features of each subgroup indicate age-specific mechanisms underlying tumor initiation. This suggests that updated clinical approaches are required to modernize treatment and improve the outcome of these infants.

## Results

### Infantile gliomas have paradoxical survival profiles

We assembled a multi-institutional infant cohort consisting of 171 samples from 150 patients diagnosed between 1986 and 2017. Histological review confirmed the diagnosis in 142/150 (94.7%) patients, of which 104 (73.2%) and 33 (23.2%) were LGG or HGG, respectively. Five cases (3.5%) displayed intermixed LGG and HGG features (Supplementary Fig. 1a, Supplementary Table 1). Young children with LGG have a worse survival when compared with older children^13,18,19^ and to clarify whether this effect can be ascribed to the demographic <1 year of age, we compared the survival of our cohort with a cohort of older children (1–18 years, SickKids LGG cohort). iLGG had a significantly worse overall survival (OS) than pediatric LGG (pLGG) (10-year OS of 71.4% (60.8–83.3%) versus 91.6% (88.5–94.8) for iLGG vs pLGG, respectively; *p* < 0.001, log-rank test Supplementary Fig. 1b). In contrast, reports have suggested better survival of iHGG as compared with older children^16^. iHGG showed a significantly better overall survival as compared with children diagnosed between the ages of 1–18 years (SickKids HGG cohort, pHGG) with a 5-year OS of 54.5% (40.0–74.2%), vs 6.6% (2.4–18.5%), respectively (*p* < 0.001, log rank test, Supplementary Fig. 1c).

### Molecular features of infantile glioma

**Fig. 1:**
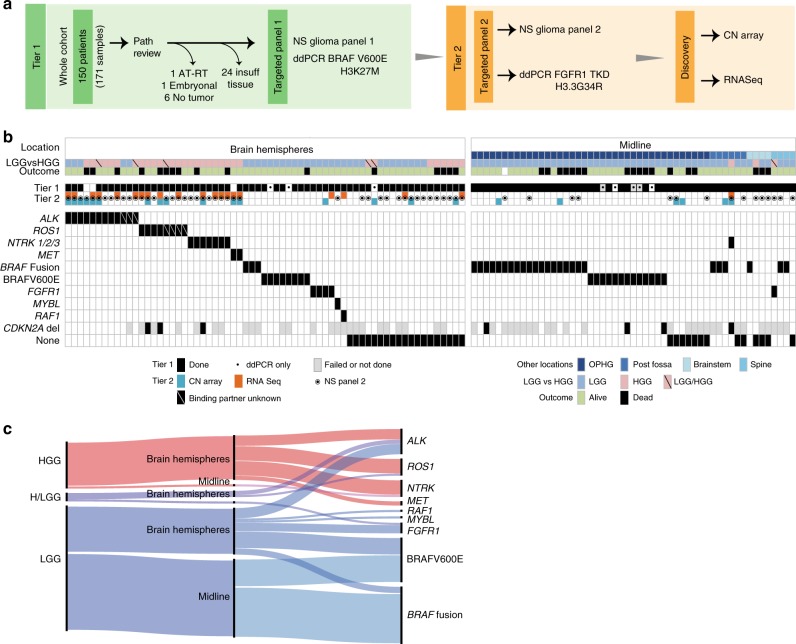
Molecular characteristics of infant gliomas. **a** The testing strategy used to identify the molecular driver of the tumor. Samples testing positive in tier 1 did not proceed to tier 2. **b** Clinical and genomic features of the infant glioma cohort highlighting the molecular alterations and clinical features associated with them including tumor location, grade and outcome. **c** Alluvial plot (https://rawgraphs.io/) showing the distribution of molecular drivers according to tumor location and histology. Red: high-grade glioma, Purple: mixed high and low-grade glioma, Blue: low-grade glioma. AT/RT: Atypical Teratoid Rhabdoid Tumors, NS: NanoString, CN: copy number, ddPCR: droplet digital PCR, HGG: high-grade glioma, LGG: low-grade glioma, OPHG: optic pathway/hypothalamic glioma

**Fig. 2:**
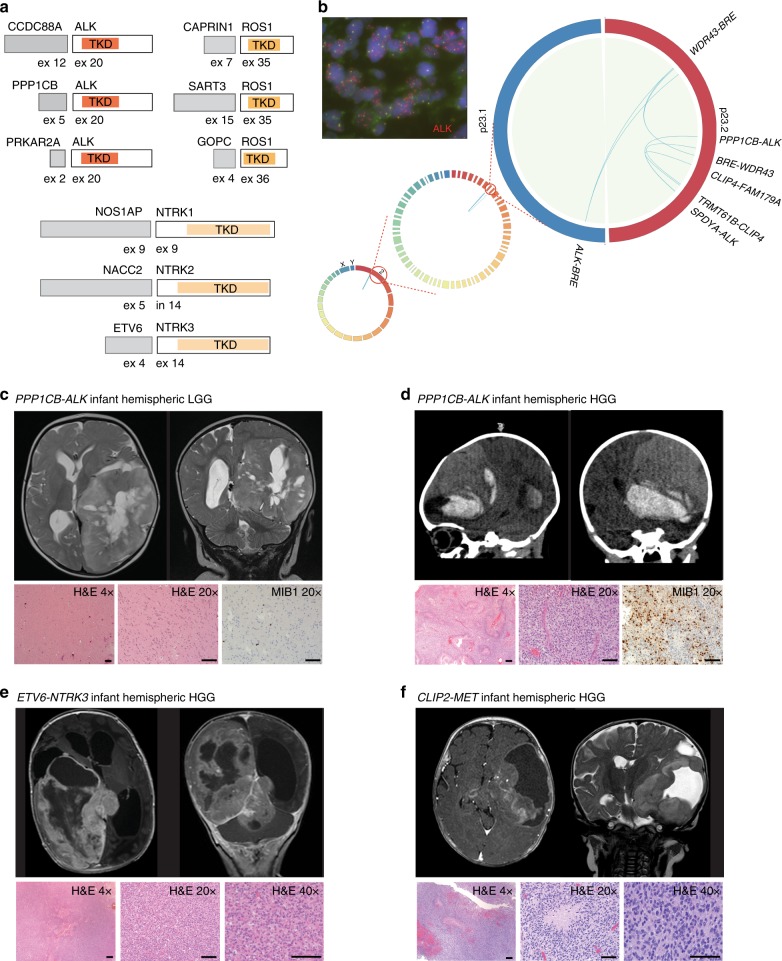
*ALK/ROS1/NTRK/MET* fused hemispheric infantile glioma. **a** Graphical depiction of the newly identified *ALK/ROS1/NTRK* fusions in infantile glioma. **b** Circos plot depicting the chromothripsis events with > 10 split-read support from total RNAseq of a *PPP1CB-ALK* tumor with progressive scaling from full chromosome set to chr 2 p23.1-p23.2 arms. FISH for *ALK* further showing evidence of *ALK* translocation and amplification in a tumor positive for *PPP1CB-ALK*. **c**, **d** Examples of *PPP1CB*-*ALK* positive tumors—an iLGG diagnosed at 10 months of age (**c**) and a congenital iHGG (**d**)—including imaging (MRI axial and coronal T2-weighted images, **c**; sagittal and coronal head CT, **d**), hematoxylin and eosin (H&E) staining, and proliferation index (MIB-1). Further examples of large hemispheric congenital iHGG harboring RTK fusions with *ETV6-NTRK3* (**e**) and *CLIP2-MET* (**f**). All images are taken at the stated magnification, scale bar = 100 μm for x20 and x40, 200 μm for ×4. TKD: tyrosine kinase domain, LGG: low-grade glioma, HGG: high-grade glioma, H&E: Hematoxylin and eosin stain

For 118/142 (83.1%) patients, sufficient tumor tissue was available for molecular characterization. We utilized a tiered molecular profiling approach combining targeted single nucleotide variant (SNV) and fusion profiling, copy number arrays and transcriptome-wide discovery strategies suitable for archival samples (Fig. 1a). *RAS/MAPK* activating alterations were the most common events (56/118, 47.5%) and primarily consisted of *KIAA1549*-*BRAF* fusions (28/118, 23.7%) and BRAFV600E mutations (21/118, 17.8%) (Fig. 1b). Additional *RAS/MAPK* pathway alterations, such as *FGFR1* fusions (*FGFR1*-*TACC1*, *n* = 3), *FGFR1*-tyrosine kinase duplications ((TKD), *n* = 2), *RAF1* fusion (*PML*-*RAF1*, *n* = 1), and *MYBL1* gain (*n* = 1) were also observed. Interestingly, *RAS/MAPK* activating events were exclusively seen in LGG and accounted for 73.6% (39/53) of alterations present in midline gliomas versus only 26.1% (17/65) of hemispheric gliomas (Fig. 1b, c). The second most common group of molecular events involved alterations in the RTK oncogenes *ALK, ROS1, NTRK*, or *MET* (30/118, 25.4%) (Fig. 1b). These events were almost exclusively observed in hemispheric tumors (29/30, 96.7%) and HGG (25/30, 83.3%) (Fig. 1b, c). *ALK*, *ROS1,* and *NTRK1/2/3* alterations led to the fusion of different 5′ binding partners with the 3′ end of the truncated RTK containing the tyrosine kinase domain **(**Fig. 2a**)**. Interestingly, *PPP1CB-ALK* fusions were detected in a region of chromothripsis on chromosome 2p (Fig. 2b) and the two most common RTK fusions—*PPP1CB-ALK* and *CCDC88A-ALK*—were found in both LGG and HGG (Fig. 2c, d; Supplementary Fig. 2). In contrast, *NTRK1/2/3* and *MET* fusions were exclusively seen in HGG (Fig. 2e, f). No IDH1R132H, H3K27M or H3G34R mutations were detected in this cohort.

### Activating *ALK* fusions are susceptible to targeted agents

**Fig. 3:**
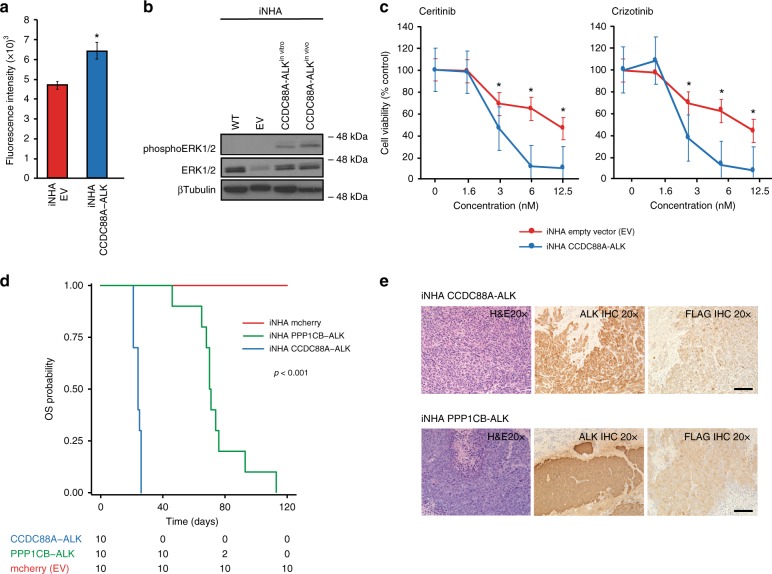
*ALK* fusions are tumorigenic and susceptible to targeted agents. **a** Proliferation of iNHA cells overexpressing *CCDC88A-ALK* compared with iNHA with empty vector (EV). Experiments were conducted in triplicates in four biological replicates (*n* = 12). Data are represented as mean with SEM, *p < 0.05, paired *t* test. **b** Western blot for total and phosphorylated ERK1/2 indicative of *MAPK* pathway activation in iNHA cells overexpressing *CCDC88A-ALK* as compared with iNHA EV. **c** Dose-response curves of iNHA expressing *CCDC88A-ALK* versus EV upon treatment with ALK inhibitors Ceritinib and Crizotinib. Experiments were conducted in triplicates in two biological replicates (n = 6). Data are represented as mean with SEM, *p < 0.05, paired *t* test. **d** In vivo orthotopic xenografts of iNHAs overexpressing *CCDC88A-ALK* and *PPP1CB-ALK* resulted in tumor formation with 100% penetrance, *p* value calculated using the log-rank test. **e** Hematoxylin and eosin (H&E) and immunohistochemistry (IHC) showing overexpression of FLAG and ALK in the intracranial xenografts. Images are taken at 20x magnification, scale bar = 100 μm. iNHA: immortalized normal human astrocytes, nM: nanomolar, OS: overall survival

Truncation of the extracellular ligand-binding domain with retention of the intracellular tyrosine kinase domain in the RTK-fusions identified suggests these are activating events **(**Fig. 2a**)**. *CCDC88A-ALK* expressing immortalized normal human astrocytes (iNHA) (Supplementary Fig. 3a, b**)** showed increased proliferation in vitro (*p* = 0.002, student’s t-test, Fig. 3a) and ERK1/2 activation (Fig. 3b). Cell viability was reduced in a dose-dependent manner when treated with ALK-inhibitors currently in pediatric clinical trials (Fig. 3c**)**. iNHAs overexpressing *CCDC88A*-*ALK* or *PPP1CB-ALK* were tumorigenic in vivo with 100% penetrance **(**Fig. 3d, e), forming glial tumors with a high MIB-1 proliferative index, pseudopalisading necrosis, focal GFAP expression, lack of synaptophysin expression and ALK overexpression (Supplementary Fig. 3c, d).

### Infantile gliomas comprise three subgroups

Analysis of the clinical features associated with each class of molecular alterations suggested that infant gliomas represent three distinct clinical/molecular groups: (1) Hemispheric, RTK-driven, (2) Hemispheric, *RAS/MAPK*-driven, and (3) Midline, *RAS/MAPK*-driven (Table 1).

**Table 1:** Summary of patient characteristics according to infant glioma subtype

Characteristic	Infant glioma subgroup		
	Group 1	Group 2	Group 3
Number	29	17	39
*Histology*			
Low Grade	5	17	39
High Grade	21	0	0
Mixed	3	0	0
*Pathology*			
Pilocytic/Pilomyxoid	0	4	27
Ganglioglioma	1	6	0
Diffuse Astrocytoma	2	1	3
Glioblastoma	15	0	0
Low-grade glioma, NOS	2	2	8
High-grade glioma, NOS	1	0	0
Other	4	4	1
*Sex*			
Male	14	11	21
Female	15	6	18
*Outcome*			
Alive	20	15	26
Deceased	9	1	12
Unknown	0	1	1
*Progression*			
Progressed	14	10	33
Stable	14	5	5
Lost to follow-up	0	1	1
Unknown	1	1	0
*Extent of surgery*			
None	1	0	1
Biopsy	5	3	13
Partial Resection	10	4	21
Gross Total Resection	12	9	3
Unknown	1	1	1
*Radiation*			
Yes	1	0	8
No	26	16	30
Unknown	2	1	1
*Chemotherapy*			
Yes	18	4	27
No	10	12	10
Unknown	1	1	2
*Age at diagnosis*			
Median (months)	2.8 (0.0–12.0)	8.3 (5.0–14.6)	7.5 (0.0–14.0)
Mean (months)	3.8 ± 3.7	9.0 ± 2.9	7.5 ± 3.4
*Progression-free survival*			
Median (years)	1.1 (0.0–17.6)	1.2 (0.1–14.2)	1.1 (0.0–17.3)
Mean (years)	2.9 ± 4.0	3.0 ± 3.8	2.8 ± 3.8
*Overall Survival*			
Median (years)	1.9 (0.0–17.7)	3.6 (0.1–16.0)	6.5 (0.1–28.5)
Mean (years)	4.4 ± 4.8	6.1 ± 5.5	7.7 ± 7.1

### Group 1: hemispheric RTK-driven

**Fig. 4:**
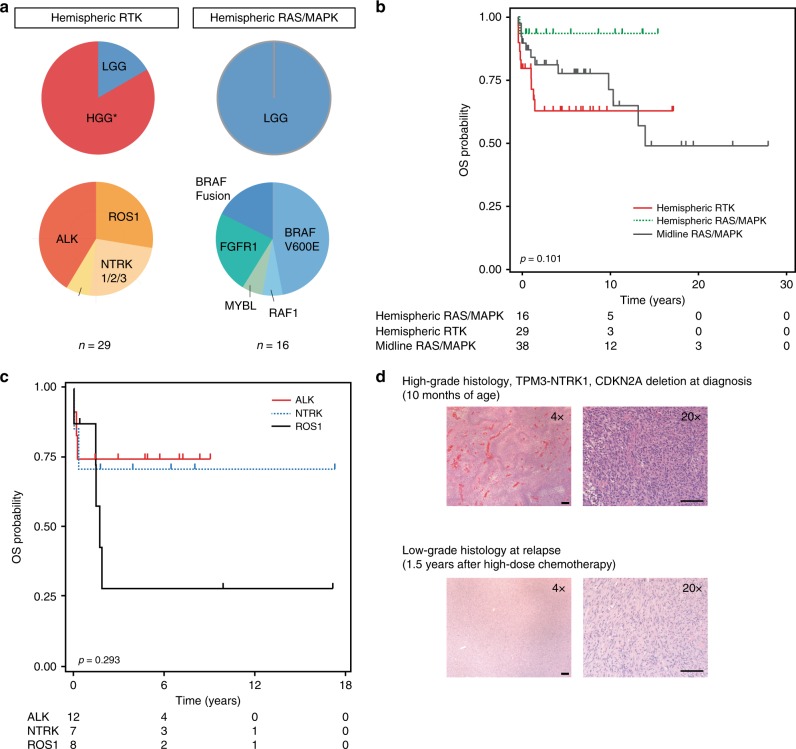
Characteristics of hemispheric glioma in infants. **a** Histological grade and molecular alterations in Group 1 Hemispheric RTK and Group 2 Hemispheric *RAS/MAPK* infant gliomas. **b** Overall survival (OS) of infants according to glioma subgroups, *p* value calculated using the log-rank test. **c** Survival of infants with hemispheric gliomas with respect to patient *ALK*, *ROS1,* and *NTRK* status, *p* value calculated using the log-rank test. **d** Hematoxylin and eosin (H&E) staining of an infant high grade glioma at diagnosis and second surgery post-chemotherapy, showing a maturing phenotype characterized by lower grade histology. Images are taken at the stated magnification, scale bar = 100 μm for x20, 200 μm for ×4. HGG: high-grade glioma, LGG: low-grade glioma

Group 1 tumors harbor *ALK/ROS1/NTRK/MET* alterations (Figs. 1b, c; 2a), and are enriched for HGG (82.8%, 24/29, *p* < 0.0001, Fisher exact test, Fig. 4a), specifically glioblastomas (15/29), and younger infants (Table 1, median age at diagnosis 2.8 months, range 0–12 months) (examples of congenital tumors in Fig. 2c–e). All LGG within this group harbored *ALK* alterations (17.2%, 5/29) while all *ROS1/NTRK/MET* alterations appeared exclusively in HGG (Figs. 2c–e, 4a). The survival of *ALK*, *ROS1,* and *NTRK* driven tumors was heterogeneous (Fig. 4b, c). Five-year OS was 53.8, 25.0, and 42.9% for *ALK, ROS1*, and *NTRK* fused tumors respectively, although the numbers in each group were small (12, 8, 7, respectively). Interestingly, when compared with *ALK*-driven HGG, low-grade *ALK* gliomas tended to be diagnosed at an older age (median = 5.0 versus 1.6 months) and showed a better clinical outcome; all patients with *ALK*-fused LGG (n = 5) were alive at a median follow-up of 5 years (range, 1.4–7.2 years), whereas 42.9% (3/7) patients with *ALK*-fused HGG were deceased at a median follow-up of 3 years (range 0.01–8.55 years). Interestingly, in two patients with *NTRK*-fused HGG that underwent a second resection post-chemotherapy, tumor from the second resection had lower grade histology, suggesting that Group 1 tumors may comprise an LGG/HGG continuum and/or have the potential to differentiate and slow their growth over time (Fig. 4d).

### Group 2: hemispheric *RAS/MAPK*-driven

**Fig. 5:**
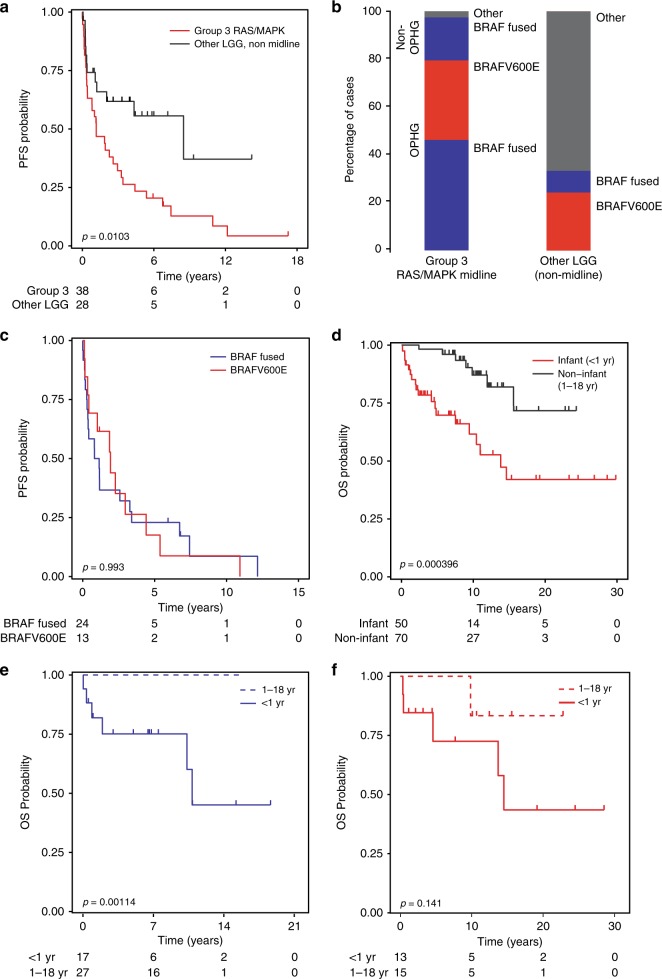
Group 3 Midline *RAS/MAPK* tumors. **a** Progression-free survival (PFS) of infants with Group 3 tumors as compared with infants with other LGG (non-midline). **b** The molecular drivers of Group 3 as compared with other infant LGG, highlighting the enrichment for *BRAF* alterations in Group 3 tumors. **c** PFS of infant Group 3 according to *BRAF* status. **d** Overall survival (OS) of infant OPHG versus non-infant (>1–18 y) OPHG in SickKids cohort. Comparison of OS of *BRAF* Fused OPHG (**e**) and BRAFV600E mutated OPHG (**f**) in infants (Group 3 of infant cohort) vs children/adolescents aged 1–18 y (SickKids cohort). All *p*-values calculated using the log-rank test. LGG: low-grade glioma

Group 2 tumors are comprised solely of hemispheric LGG and represent 26.1% (17/65) of hemispheric gliomas in infants. Group 2 tumors more frequently had non-*BRAF RAS/MAPK* activating events when compared with Group 3 tumors (35.3% vs 2.6%, respectively) (Figs. 4a, 5b). Group 2 tumors have the best outcome of the three subgroups with 10-year OS of 93.3% (81.5–100%) (Fig. 4b), were more readily resected (52.9%, 9/17 gross-total resection (GTR)) versus midline (7.7%, 3/39), and were less likely to require a second line of treatment (0% and 23.5% received radiation or chemotherapy, respectively) (Table 1).

### Group 3: midline *RAS/MAPK*-driven

Three quarters (39/53) of all midline infantile gliomas were *RAS/MAPK* driven, 97.4% of which (38/39) harbored canonical *BRAF* alterations. Group 3 infantile gliomas were histologically LGG, primarily consisting of pilocytic astrocytoma (69.2%, 27/39, Table 1). Survival of infant patients with Group 3 tumors was significantly worse with 5-year progression-free survival (PFS) of 23.4% (12.9–42.5%) compared with 55.6% (38.5–80.3%) for infants with other LGGs (non-midline) (*p* = 0.01030, log-rank test, Fig. 5a). Group 3 tumors consisted primarily of optic pathway hypothalamic glioma (OPHG) (31/39, 79.5%) and *RAS/MAPK-*activation was almost exclusively due to *BRAF* alterations (Fig. 5b). Importantly, despite half of all OPHGs in this study being driven by BRAFV600E, no non-OPHG group 3 tumors harbored this mutation (Fig. 5b). No difference in survival was observed between *BRAF* alterations in Group 3 tumors (Fig. 5c). When compared with OPHG in older patients, infants with OPHG had a poorer outcome with 10-year OS of 57.7% (42.8–77.9%) compared with 87.1% (76.8–98.7%) in infant vs SickKids OPHG 1–18 y cohort, respectively (*p* < 0.001, log-rank test, Fig. 5d). Age at diagnosis (infant vs. non-infant) was the only significant predictor of OS both on univariate (HR = 12.839, *p* = 0.001) and in a multivariate analysis that included sex, extent of resection, *BRAF* fusion and BRAFV600E status, chemotherapy and radiation (HR = 27.084, *p* = 0.001, Supplementary Table 2). The 5-year OS was 75.1% (56.6–99.7%) for infant *BRAF*-fused OPHG and 72.5% (49.5–100%) for infant BRAFV600E OPHG (Fig. 5e, f). This is in stark contrast to older children where long-term survival of patients with *BRAF*-fused OPHG is excellent (5-year OS 100%, *p* = 0.00114, log-rank test, Fig. 5e). A similar trend was seen in BRAFV600E OPHG compared between infants and older children, albeit not statistically significant (*p* = 0.1410, log-rank test, Fig. 5f). Interestingly, despite the striking differences in outcome, infant and pediatric gliomas clustered more according to location rather than molecular alteration or outcome on methylation analysis (Supplementary Fig. 4).

## Discussion

**Fig. 6:**
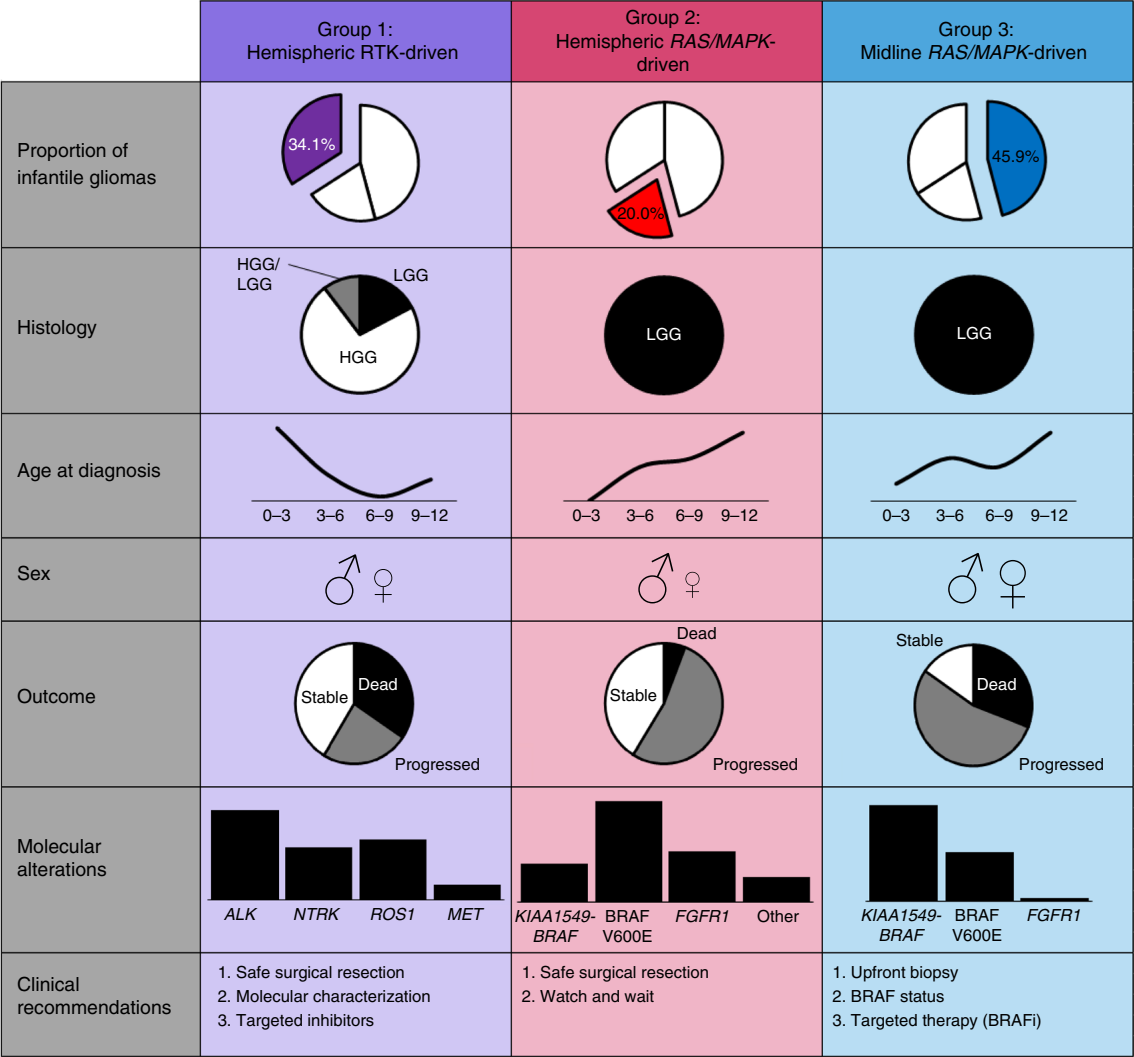
Graphical summary of the three infant glioma subgroups. HGG: high-grade glioma, LGG: low-grade glioma

In this study we comprehensively characterize the landscape of genetic drivers and their clinical impact, revealing 3 subgroups of infant glioma (Fig. 6). Group 1 tumors are enriched for *ALK/ROS1/NTRK/MET* fusions, alterations analogous to those detected in adult carcinomas such as non-small cell lung cancer^20,21^ and colorectal cancer^22^. Despite similar conservation of the tyrosine kinase domain and region of breakpoints, for most cases the binding partners identified in infant gliomas differ from those in other malignancies. Interestingly, *ETV6-NTRK3* can also be detected in other congenital tumors (congenital mesoblastic nephroma and congenital fibrosarcoma), suggesting a common age-specific mechanism. With the exception of *NTRK* fusions, which were previously shown to be enriched in non-brainstem infant HGG^23^, these alterations have been rarely reported in gliomas and this study provides a comprehensive explanation for the isolated case reports of *ALK*^24,25^ and *ROS1*^26^ fusions in pediatric glial tumors. Indeed, these alterations are recurrent and define, together with *NTRK*, Group 1 hemispheric infant gliomas. Examination of the clinical data in these cases reveals several interesting facts: (1) their overall survival is good compared with that of older children with HGG and if they survive past two years, almost none progress further; (2) cases where a second surgery was done post-chemotherapy show differentiation and decreased proliferation of the tumor; and (3) cases with LGG histology tend to occur in older infants. Taken together, these observations suggest the capacity for differentiation over time in Group 1 tumors, perhaps, as in other pediatric gliomas, through oncogene induced senescence^27–30^. Alternatively, as seen in neuroblastoma, a common infant tumor that harbors *ALK* alterations, inherent maturation (also a part of normal development) may explain the morphological and clinical “maturation” of some iHGG into iLGG^31–33^. This has important implications for our therapeutic approach as it suggests that if we can use non-morbid treatment options, which may include targeted kinase inhibitors, to get them through the rapid growth phase of their tumor, their long-term outlook may be positive. Since infantile gliomas are mostly single-driver tumors, unlike adult lung and colorectal cancers, they are particularly suitable for precision-medicine treatment approaches. Several ALK inhibitors have either already shown efficacy or are in clinical trials for *ALK*-driven tumors in children, including Crizotinib^34^ and Ceritinib, and the newer generation inhibitors with enhanced blood-brain barrier penetration Lorlatinib and Ensartinib. The NTRK inhibitor Larotrectinib has also shown antitumor activity in pediatric patients with *NRTK*-fused tumors regardless of age or histology^35–37^. For example, in the NAVIGATE Phase 2 trial, Larotrectinib treatment resulted in a significant decrease in tumor volume in a 35-year-old woman with glioblastoma^38^. In the STARTRK1 trial, Drilon et al.^39^ report a pontine astrocytoma harboring an *NTRK* fusion that showed tumor volume reduction upon treatment with Entrectinib, a tyrosine kinase inhibitor known to target *NTRK*, *ALK* and *ROS1*. These encouraging results have led to a current phase I/Ib study being conducted in pediatrics to evaluate Entrectinib in primary CNS tumors (NCT02650401), which includes *NTRK*, *ROS1*, and *ALK* fused tumors. Results thus far are promising^40^.

Group 2 hemispheric *RAS/MAPK* tumors have an excellent long-term survival and often require only surgery, suggesting that a safe resection and a careful “watch and wait” postsurgical strategy is appropriate for these patients. Group 3 represents midline LGG enriched for *RAS/MAPK* alterations. The lack of HGG histology, such as that observed in the pons or thalami of older children, and the lack of histone mutations in this age group suggest distinct tumor- and/or host-related factors underlying tumor development. In older children, *BRAF* fused-tumors tend to have favorable outcome^5,41,42^ and a good response to conventional therapy. Strikingly, most Group 3 tumors, especially OPHG, progressed regardless of *BRAF* fusion or mutation status. The poor outcome of *BRAF*-fused midline tumors in infants is surprising and in stark contrast to the biological behavior of similar tumors in older children. This disparity may be related to age-specific genetic, tumor or microenvironment factors that are, at this point, poorly understood. As such, there is little or no room for “watch and wait” and a biopsy should be performed upfront to ascertain *BRAF* status and systemic therapy initiated readily thereafter. Given the multiple progressions typically observed with conventional chemotherapy and the encouraging results of targeted BRAF/MEK inhibitors in pLGG^43,44^, these patients should be prioritized for targeted therapies early after initial diagnosis.

Whereas future studies will certainly further characterize infant gliomas, our study broadens our understanding of cancers early in life and emphasizes the need for age-specific diagnostic and treatment guidelines. Our data have immediate therapeutic implications and provide a rationale for early molecular pathology consultation, prospective collection of clinical information and inclusion of infants in upfront clinical trials with targeted inhibitors.

## Methods

### Patient samples

Tumor specimens and clinical information were collected with informed or waived consent in accordance to protocols approved by the Research Ethics Board at the Hospital for Sick Children (Toronto, ON.) and each of the respective participating institutions. For patients diagnosed at the Hospital for Sick Children (SickKids) and older than 18 years at the time of clinical data collection, survival information was extracted from the Pediatric Oncology Group of Ontario Network Information System (POGONIS)^45^. A central pathology review was completed to ascertain tumor content and confirm the diagnosis of the specimen where applicable. As only selected slides were available for central review from participating institutions, histological grading rendered at the original institution was used.

### Nucleic acid extraction

DNA was extracted from 3–5 10 µm thick scrolls obtained from formalin-fixed paraffin embedded (FFPE) tissue either shaved from the original block or scraped from unstained slides. The extraction was completed with the QIAamp DNA FFPE Tissue Kit (Qiagen, Valencia, CA). If available, 10–20 mg of fresh frozen tissue rather than FFPE was used for extraction with the DNeasy Blood and Tissue Kit (Qiagen, Valencia, CA). DNA was quantified with the Qubit Fluorometer V2.0 using the dsDNA Broad Range Assay Kit (Thermo Scientific, Waltham, MA). All assay kits and quantification methods were used according to the manufacturer’s guidelines.

RNA was extracted from 3 to 5 10 µm thick scrolls obtained from FFPE either shaved from the original block or scraped from unstained slides. Extraction was completed using the ExpressArt FFPE Clear RNA extraction kit (Amsbio, Cambridge, MA). If available, 10–20 mg of fresh frozen tissue rather than FFPE was used for extraction using the RNeasy Mini Kit (Qiagen, Valencia, CA). RNA was quantified with the Qubit Fluorometer V2.0 using the RNA Broad Range Assay Kit (Thermo Scientific, Waltham, MA). All assay kits and quantification methods were used according to the manufacturer’s guidelines.

### Droplet digital PCR

Droplet digital PCR was conducted according to manufacturer guidelines. Samples consisted of 1X ddPCR Supermix for probes (no dUTP) (Bio-Rad, Hercules, CA), 900 nM of HPLC-purified forward and reverse primers, 250 nM of target-specific mutant and wild type locked-nucleic acid (LNA) probes, and 10–20 ng of genomic DNA in 20 µl of total volume. Each reaction was mixed with 70 μl of Droplet Generation Oil (Bio-Rad, Hercules, CA) and partitioned into a minimum of 10,000 droplets (range 10,000–15,000) on the QX200 droplet generator (Bio-Rad, Hercules, CA). 40 μl of the resultant droplets were transferred to a 96-well plate and sealed prior to polymerase chain reaction (PCR) amplification. PCRs were performed on a T1000 Thermal Cycler (Bio-Rad, Hercules, CA) and cycling conditions were as follows unless otherwise specified: 95 °C for 10 min, 39 cycles of 94 °C for 30 s and 55 °C for 60 s (with a 2 °C s^−1^ ramp rate), 98 °C for 10 min, and held at 4 °C. Following amplification, fluorescent intensity was measured with the QX200 Droplet Reader (Bio-Rad, Hercules, CA) and data analysis performed with the QuantaSoft droplet reader software (Bio-Rad, Hercules, CA). Positive and negative droplet populations were detected on two-dimensional graphs and the absolute transcript levels were computed as a percent of the total gene copy. All samples were run in duplicate to ensure validity. Samples were considered positive if a minimum 1% mutant allele frequency was detected in both duplicate runs and a minimum threshold of 50 total droplets containing fluorescent signal were detected. The following assay IDs were used (Bio-Rad, Hercules, CA):

1. PrimePCR ddPCR mutation assay BRAF WT/V600E for p.V600E, Human (unique assay ID: dHsaCP2000027/28).
2. PrimePCR ddPCR mutation assay H3F3A WT/K28M for p.K28M, Human (unique assay ID: dHsaCP2500510/11).
3. PrimePCR ddPCR mutation assay H3F3A WT/G35R for p.G35R, Human (unique assay ID: dHsaMDS720957813).
4. Prime PCR ddPCR copy number assay *CDKN2A*, Human unique assay ID: dHsaCP1000581) and reference prime PCR ddPCR copy number assay *APB31* (unique assay ID: dHsaCP2500348). A known homozygous deleted cell line was used as a zero-copy control, whereas an Ontario Population Genomics Platform healthy control sample (ID: 85751) obtained from The Center of Applied Genomics at SickKids was used as a two-copy control. Samples that showed < 1.2 copy number value as calculated from the total target and reference event number were considered deleted.
5. FGFR1 TKD is a custom assay design^46^, primers and probes were designed by Integrated DNA Technologies (IDT) as follows: FGFR1 Exon 8 Forward: 5′-TTCCCTTGCTCTGCGTCTCT-3′, FGFR1 Exon 8 Reverse: 5′-TCCATCTCTTTGTCGGTGGTATT-3′, FGFR1 Exon 8 HEX-probe: 5′ HEX-TTGCTTCCGTTGTCTCTTCTAGACTGCTGG-3′, FGFR1 Exon 16 Forward: 5′-CACTGCCCTGGGTAGAGGATT-3′, FGFR1 Exon 16 Reverse: 5′-ACAGGAGCACCCCGAAAGA-3′, and FGFR1 Exon 16 FAM-probe: 5′ FAM-CTCTAACACCCTGTGGCTCTCCGCC-3′. PCR cycling conditions were as follows: 95 °C for 10 min, 39 cycles of 94 °C for 30 s and 55 °C for 60 s (with a 2 °C s^−1^ ramp rate), 98 °C for 10 min, and a 15 °C hold. A ratio value of 1.125 for exon 16 relative to exon 8 were called duplicated.

### NanoString nCounter

Panel 1: Samples were tested for fusion gene expression with the NanoString nCounter (NanoString, Seattle, WA) Low Grade Glioma Panel 1^47^. In all, 200–500 ng of RNA was mixed with panel specific CodeSet (Low Grade Glioma Panel 1) and allowed to hybridize overnight for 20 h. CodeSet/RNA complexes were then purified and immobilized onto the nCounter cartridge system (NanoString, Seattle, WA). The nCounter cartridge was then scanned at 555 fields of view on the nCounter Digital Analyzer (NanoString, Seattle, WA) to identify the unique fluorescent signatures (barcode) associated with each CodeSet probe. The barcodes are counted and background adjusted with a Poisson correction based on the negative control spikes included in each run. This was followed by a technical normalization using the four housekeeping transcripts included in each run (*ABCF1*, *ALAS1*, *CLTC*, and *HPRT1*). Data is viewed using a box plot and the extreme statistical outlier (3X the interquartile range (IQR)) method was used to detect the presence of an expressed fusion.

Panel 2: To account for an evolving knowledge of fusions described in gliomas, a second NanoString fusion panel was designed. The fusion targets included on this panel are listed in Supplementary Table 3. In addition to fusion targets, three reporter targeting systems were also included targeting *ALK*, *ROS1,* and *NTRK2*. These reporter systems work by adding multiple sequence tags prior to and after the exons of well-defined breakpoint hotspots. In the event of a breakpoint, the reads from the nCounter appear significantly different between adjacent sequence tags, allowing for the identification of a likely fusion event with an unknown partner. Samples were tested for fusion gene expression with the NanoString nCounter Low Grade Glioma Panel 2 as described above. CodeSet probe sequences for Panel 1 and 2 are proprietary, but available from NanoString Technologies (Seattle, WA) and the Hospital for Sick Children upon request.

### Fluorescent in situ hybridization

Fluorescent in situ hybridization (FISH) analysis was performed on formalin fixed paraffin embedded 4-μm tumor sections using a dual color breakapart probe for the *ALK* gene (Empire Genomics, Buffalo, NY). Slides were baked overnight to fix the section to the slide and were pretreated by using a paraffin pretreatment kit (Abbott, Chicago, IL). Sections were dehydrated before slide/probe co-denaturation on thermobrite (Intermedico, Markham, ON). Denaturation conditions used for paraffin-embedded slides/probes were as follows:

1. 83 °C for 7 min
2. 37 °C overnight

Slides were washed in 0.4x Saline-sodium citrate(SSC)/0.3% NP-40 at 65 °C for 30 s, followed by 2x SSC/0.1% NP-40 at room temperature for 30s. Slides were counterstained with DAPI. Nuclei were analyzed by using an Axioplan2 epifluorescence microscope (Zeiss, Jena, Germany). Images were captured by an Axiocam MRm Camera (Imaging Associates, Bicester, United Kingdom) and analyzed by using an imaging system with Isis Software (Version 5.1.110; MetaSystems, Boston, MA).

### Copy number analysis

The OncoScan FFPE Assay Kit (Affymetrix, Santa Clara, CA, USA) was used to assess copy number and loss of heterozygosity events in selected samples that remained uncharacterized by the targeted methods described above. Samples for this assay were sent to the Genome Quebec Innovation Centre for completion of the analysis. The OncoScan FFPE Assay Kit (Affymetrix, Santa Clara, CA, USA) was used according to manufacturer’s specifications and sample preparation, including digestion, labelling, quality checks, hybridization, and scanning was performed at the Genome Quebec Innovation Centre. Data was analyzed using the Chromosome Analysis Suite (ChAS) (ThermoFisher Scientific, CA, USA) and copy number calls based on normalized data.

### Targeted RNA sequencing

TruSight Sequencing Panel: Samples with sufficient RNA for sequencing had their total RNA constructed into RNA-sequencing libraries using the Illumina TruSight RNA Pan-Cancer Panel Kit (Illumina, San Diego, CA), following the manufacturer’s guidelines. cDNA generation was completed by random priming during first and second strand synthesis, followed by 3′ end adenylation. Sequencing adapters were then ligated to the fragments to allow for amplification of the cDNA followed by a validation step to ensure proper adapter ligation. Samples were then hybridized to specific target probes used to enrich for cancer-associated genes outlined in the manufacturer’s documentation. Paired-end RNA-sequencing was performed using the NextSeq 550 (Illumina, San Diego, CA), sequencing platform. Raw sequencing data was converted to fastq files and analyzed using the BaseSpace application (Illumina, San Diego, CA) with RNA-Seq Alignment V.1.0.0. Variant calling was completed in BaseSpace using the Isaac Variant Caller^48^ while structural rearrangements were identified using Manta^49^ and TopHat^50^.

### Whole-transcriptome sequencing

Samples with sufficient RNA quality and quantity were sent for whole transcriptome sequencing at The Center for Applied Genomics (Hospital for Sick Children, Toronto, ON). Library preparation was completed using the TruSeq RNA Library Prep Kit v2 (Illumina, San Diego, CA) using the rRNA depletion kit RiboZero Gold (Illumina, San Diego, CA) according to the manufacturer’s specifications. Paired-end sequencing was performed on the Illumina HiSeq 2500 platform. STAR^51^ was used to align the raw sequencing data to genome reference “Homo sapiens UCSC hg19”. Fusion events were called using four fusion callers: defuse^52^, tophat^50^, ericscript^53^, and fusionmap^54^.

### DNA methylation analysis

Methylation profiling was completed at the microarray centre at the Centre for Applied Genomics at the Hospital for Sick Children (Toronto, Canada). Bisulphite conversion was completed using the EZ DNA Methylation kit (Zymo Research) according to the manufacturerʼs guidelines. Genome-wide DNA methylation patterns were analyzed using the HumanMethylation450 BeadChip platform according to manufacturer specifications (Illumina, San Diego, CA). Raw data underwent quality control and pre-processing using the R package “minifi”^55^ and normalized using the R package “noob”^56^. Probes with a SNP at or near the CpG, plus those on the X and Y chromosomes were removed. t-SNE plots were completed using the R package “t-SNE”^57^. Raw.idat files are available at at the GEO wesbite under the ascension code GSE135017.

### Generation of preclinical models

In vitro: immortalized (TERT/E6/E7) normal human astrocytes (iNHA) were a gift from Dr. Pieper^58^ and maintained in culture in DMEM supplemented with 10% Fetal Calf Serum and 1% Penicillin/Streptomycin. FLAG-tagged DNA sequences for the gene fusions *CCDC88A*-*ALK* and *PPP1CB*-*ALK* were cloned into pLVX-IRES-mcherry by GenScript USA. Stable lines were generated by lentiviral transduction and mcherry-positive cells selected by FACS sorting (Supplementary Fig. 3). Proper integration was confirmed via PCR analysis using the following primer sequences: *CCDC88A-ALK* forward: 5′-TTGGCTGGGAACTGGAACAG-3′, *CCDC88A-ALK* reverse: 5′-CAGCAAAGCAGTAGTTGGGG-3′, *PPP1CB-ALK* forward: 5′-GATTGTCACCAGACCTGCA-3′, *PPP1CB-ALK* reverse: 5′-CGGAGCTTGCTCAGCTTGTA-3′ mCherry forward: 5′-CGAGGAGGATAACATGGCCATC-3′, mCherry reverse: 5′-CATCACGCGCTCCCACTTGAAG-3′, RPPH1 forward 5′-TGTCACTAGGCGGGAACACC-3′, and RPPH1 reverse: 5′-CTCCGCCCTATGGGAAAAAG-3′. Cell lines are available upon request.

In vivo: all in vivo studies were reviewed and accepted by the Animal Care Committee at The Centre for Phenogenomics (Toronto, ON), an affiliate of the Hospital for Sick Children (Toronto, ON). For the intracranial orthotopic in vivo model, 200,000 iNHA mcherry EV, *CCDC88A*-*ALK* or *PPP1CB*-*ALK* cells were injected in the brain hemispheres of age (8–10 weeks) and sex-matched NOD/scid/gamma (NSG) mice randomly assigned to either a control or experimental group. Animals were independently monitored by a third party and euthanized at humane endpoints when physiological signs of a brain tumor (hunched posture, scruffy appearance, weight loss, etc.) were detected or at 6 months post injection for the control group. CNS samples were collected at endpoint and evaluated histologically for tumors by The Centre for Phenogenomics (Toronto, ON.) histology core.

### In vitro proliferation assay

Cells were seeded at 10,000 cells/well in a 96-well plate and allowed to adhere for 48 h. 20 µl of Alamar Blue (Thermo Fisher, CA., USA) was added to each well and the plates incubated at 37 °C and 5% CO2 for 4 h. The fluorescence intensity was measured using a Spectramax Gemini plate reader (Molecular Devices, San Jose, CA, USA) using an excitation wavelength of 530 nm and an emission wavelength of 580 nm. Analysis was completed by normalizing intensity values against wells containing media alone. Data was represented as the mean of each condition. No detectable batch effect was observed.

### In vitro drug dose assay

Ceritinib (LDK-378) and Crizotinib (PF-02341066) were purchased from Selleckchem.com and prepared according to the manufacturer’s guidelines. Drugs were diluted in DMSO to the defined concentrations. Cells were seeded at 5,000 cells/well in a 96-well plate and allowed to adhere for 24 h. After 24 h, the appropriate drug concentration was added to each well and the plates incubated at 37 °C and 5% CO2 for 48 h. The fluorescence intensity was measured using a Spectramax Gemini plate reader (Molecular Devices, San Jose, CA, USA) using an excitation wavelength of 530 nm and an emission wavelength of 580 nm. Analysis was completed by normalizing intensity values against wells containing media alone. Data was represented as mean of each condition. No detectable batch effect was observed.

### Immunohistochemistry

ALK and FLAG immunostaining was performed using 10 μm-thick sections of the tumor samples post de-parafinization. Antigen retrieval was performed in a citrate buffer (pH 6.0) for 5 min prior to peroxidase quenching with 3% hydrogen peroxide (H2O2) in PBS for 10 min. The sections were then washed in water and pre-blocked with a normal goat or horse serum for 1 h. Next, the tissue sections were incubated overnight at 4 °C in primary antibody:

1:50 anti-ALK rabbit monoclonal primary antibody, Clone D5F3, Cell Signaling Technology (Danvers, MA, USA).

1:50 anti- FLAG (Monoclonal anti-FLAG M2 antibody, F1804, Sigma-Aldrich (St. Louis, MO, USA).

MIB-1, Synaptophysin and GFAP immunohistochemistry was performed on a Benchmark Ventana Machine (Tucson, AZ) using the Optiview detection kit (Tucson, AZ). CC1 was used for heat retrieval for 40 min. Tissue sections were incubated with primary antibody for thirty-six minutes:

RTU anti-MIB-1 (mouse monoclonal primary antibody, GA626, ready-to-use, Dako Omnis, Santa Clara, CA, USA).

RTU anti-Synaptophysin (mouse monoclonal primary antibody, GC202, ready-to-use, Dako Omnis, Santa Clara, CA, USA).

RTU anti-GFAP (rabbit polyclonal primary antibody, GA524, ready-to-use, Dako Omnis, Santa Clara, CA, USA).

After washing the sections with PBS, they were incubated with secondary antibodies (1:100) for 1 h. The Mouse on Mouse Polymer IHC kit (Abcam, Cambridge, UK) was used via the manufacturer guidelines prior to image acquisition to mitigate cross-reactivity and improve sensitivity for antibodies raised in mice. Finally, the sections were developed with diaminobenzidine tetrahydrochloride substrate for 10 min, and counterstained with hematoxylin. Pictures were obtained using a Nikon E600 microscope (Nikon, Canada).

### Western blotting

Total cellular proteins were extracted with 2X SDS lysis buffer containing 1 M tris (pH 7.4), 0.5 M EDTA, 10% SDS, and glycerol. The proteins were separated on a sodium dodecylsulfate-polyacrylamide gel (Novex WedgeWell 4–20% Tris-Glycine Gel, Invitrogen) electrophoresis (SDS-PAGE), which were then transferred onto nitrocellulose membranes. The nitrocellulose membranes were incubated with the appropriate primary antibodies suspended in 5% albumin blocking solution, followed by the secondary antibodies conjugated to horseradish peroxidase. Antibody binding was detected with Pierce enhanced chemiluminescence reagent western blotting substrate (Thermo Scientific Rockford, USA). Antibodies used were 1:5000 ALK (3633), 1:1000 p-ERK1/2 (9101), 1:5000 Total ERK 42/44 (9102), and 1:10,000 tubulin (2144) purchased from Cell Signaling Technology (Danvers, MA, USA). 1:5000 Anti-Flag (F1804) was purchased from Sigma-Aldrich (St. Louis, MO, USA).

### Immunocytochemistry

iNHA cells plated on coverslips were fixed with 4% PFA for 10 min and permeabilized with 0.2% Triton-X for 15 min. Coverslips were blocked (1% BSA, 2.5% Donkey serum, 0.05% Tween-20) for 1 h and probed overnight for FLAG (Sigma, F1804, 1:100) and ALK (Cell Signaling Technology, 3633, 1:200). FLAG and ALK antibodies were subsequently labelled with FITC and TRITC labelled antibodies and mounted (Vector Laboratories, Vectashield, H-1200). Images were acquired using Di8 spinning disk confocal microscope (Leica Microsystems) (40x objective lens) and Volocity software (Quorum Technologies).

### Statistics

Statistical analyses were performed using R version 3.5.0 and R Commander Version 2.4–4 with the plugins “Survival” (version 1.2–0), “KMggplot2” (version 0.2–5) and “Plot by Group” (version 0.1-0). PFS was defined as the time between diagnosis and tumor progression requiring a change in clinical management. OS was defined as the time from diagnosis until death or last follow up for the patients still alive. Estimations of survival were calculated using the Kaplan-Meier method and log rank test, p values below 0.05 were considered significant. 5 and 10 year survival is reported as a percentage with 95% confidence intervals. Univariate and multivariate analysis was performed using SPSS v25 (IBM Corporation). This was done using a univariate or multivariate Cox proportional hazards model and significance testing (*α* = 0.05) based on the Wald test.

### Source data

Uncropped and unedited gels and blots are contained within Supplementary File Source Data. Raw clinical features used for survival plots and prognostic analysis are also included in this file.

### Reporting summary

Further information on research design is available in the Nature Research Reporting Summary linked to this article.

## Supplementary information

Supplementary Information

Reporting Summary

Source Data

## Data Availability

The targeted and whole transcriptome sequencing data sets have been deposited in the European-Genome-phenome Archive under accession code EGAS00001003714. The methylation data is available from the GEO website under the accession code GSE135017. All the other data supporting the findings of this study are available within the article, its supplementary information files and from the corresponding author upon reasonable request. A reporting summary for this article is available as a Supplementary Information file.
