# Supplementary material for: Alterations in ALK/ROS1/NTRK/MET drive a group of infantile hemispheric gliomas
Source: Nat Commun. 2019 Sep 25;10:4343. doi: 10.1038/s41467-019-12187-5 (PMC6761184; doi:10.1038/s41467-019-12187-5)
Supplement: Supplementary file 1 — Supplementary Information [file 41467_2019_12187_MOESM1_ESM.pdf]

Supplementary Information

Alterations in ALK/ROS1/NTRK/MET drive a group of infantile hemispheric gliomas

Guerreiro Stucklin *et al*.

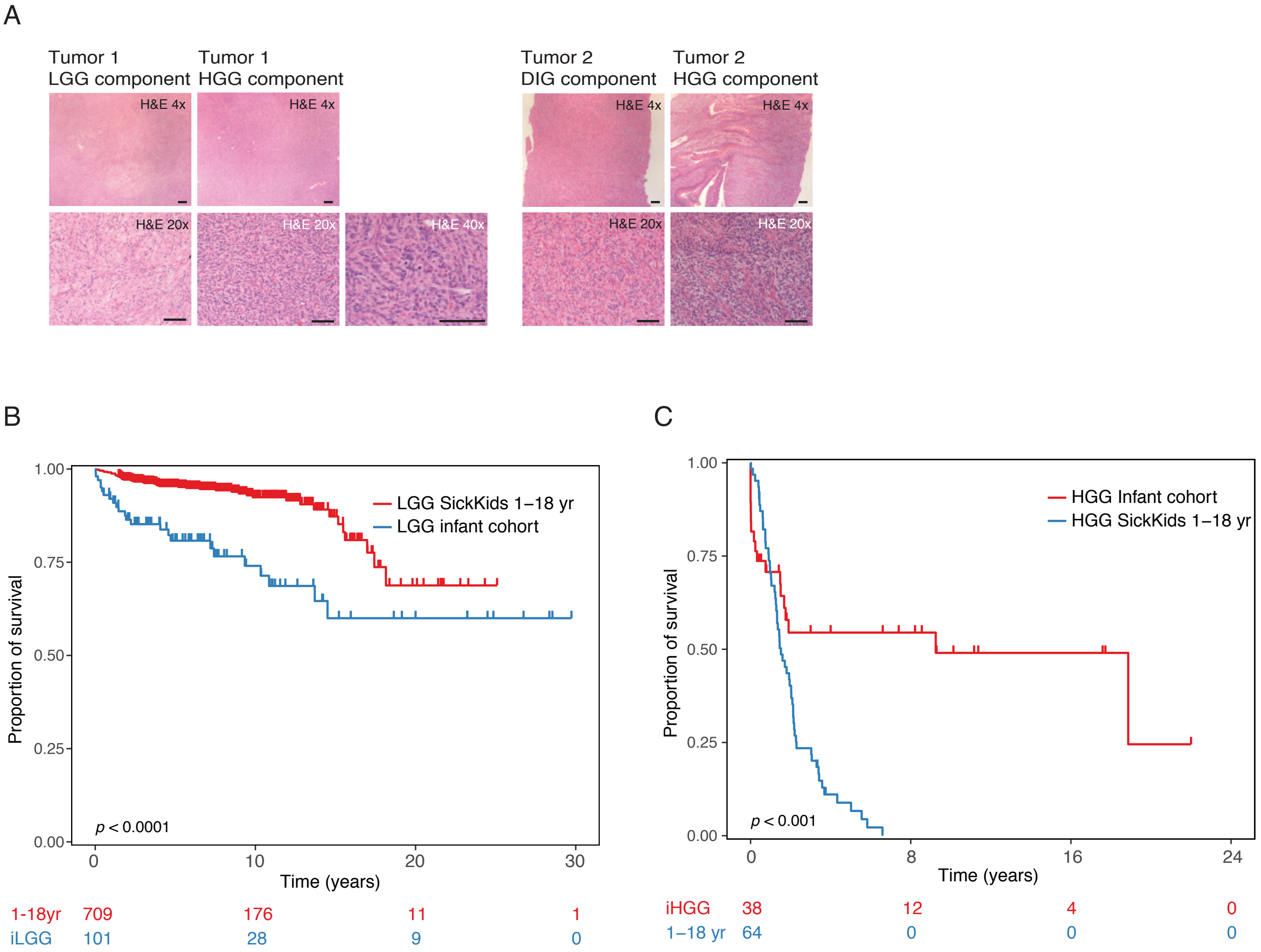
A
Tumor 1
LGG component
Tumor 1
HGG component
Tumor 2
DIG component
Tumor 2
HGG component
H&E 4x
H&E 4x
H&E 4x
H&E 4x
H&E 20x
H&E 20x
H&E 40x
H&E 20x
H&E 20x
B
1.00
0.75
0.50
0.25
0.00
Proportion of survival
LGG SickKids 1–18 yr
LGG infant cohort
p < 0.0001
0
10
20
30
Time (years)
1-18yr 709 176 11 1
iLGG 101 28 9 0
C
1.00
0.75
0.50
0.25
0.00
Proportion of survival
HGG Infant cohort
HGG SickKids 1–18 yr
p < 0.001
0
8
16
24
Time (years)
iHGG 38 12 4 0
1–18 yr 64 0 0 0

Supplementary Figure 1. (A) Examples of 2 tumors with both LGG and HGG histological components as highlighted by Hemotoxylin and Eosin staining (H&E). All images are taken at the stated magnification, scale bar = 100 μM, 200 μM for 4x. (B) Overall survival (OS) of infants (infant cohort, <1 year of age) and children/adolescents aged 1-18 years (Sick Kids cohort) with LGG, p value calculated using the log-rank test. (C) OS of infants (infant cohort, <1 year of age) and children/adolescents aged 1-18 years (Sick Kids cohort) with HGG, p value calculated using the log-rank test.

LGG: low-grade glioma HGG: high-grade glioma

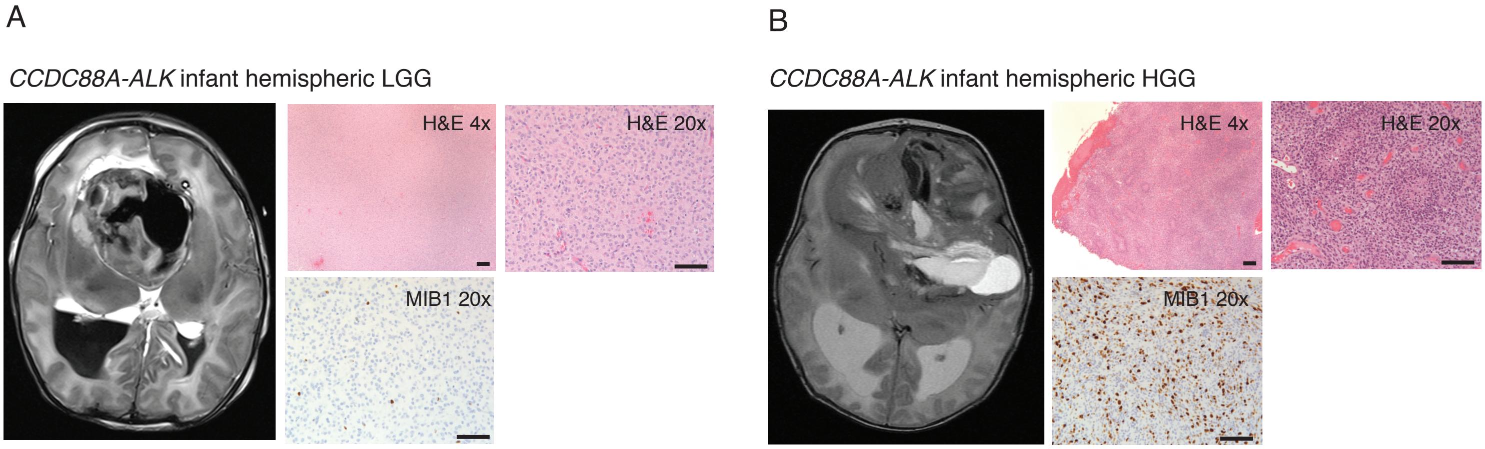
A
CCDC88A-ALK infant hemispheric LGG
H&E 4x
H&E 20x
MIB1 20x
B
CCDC88A-ALK infant hemispheric HGG
H&E 4x
H&E 20x
MIB1 20x

Supplementary Figure 2. Examples of CCDC88A-ALK positive infant gliomas with (A) low-grade glioma (LGG) histology and (B) high-grade glioma (HGG) histology. Panels include hemotoxylin and eosin (H&E) staining, proliferation index (MIB1) and FISH for ALK break apart probes. Images are taken at the stated magnification, scale bar = 100 μM, 200 μM for 4x.

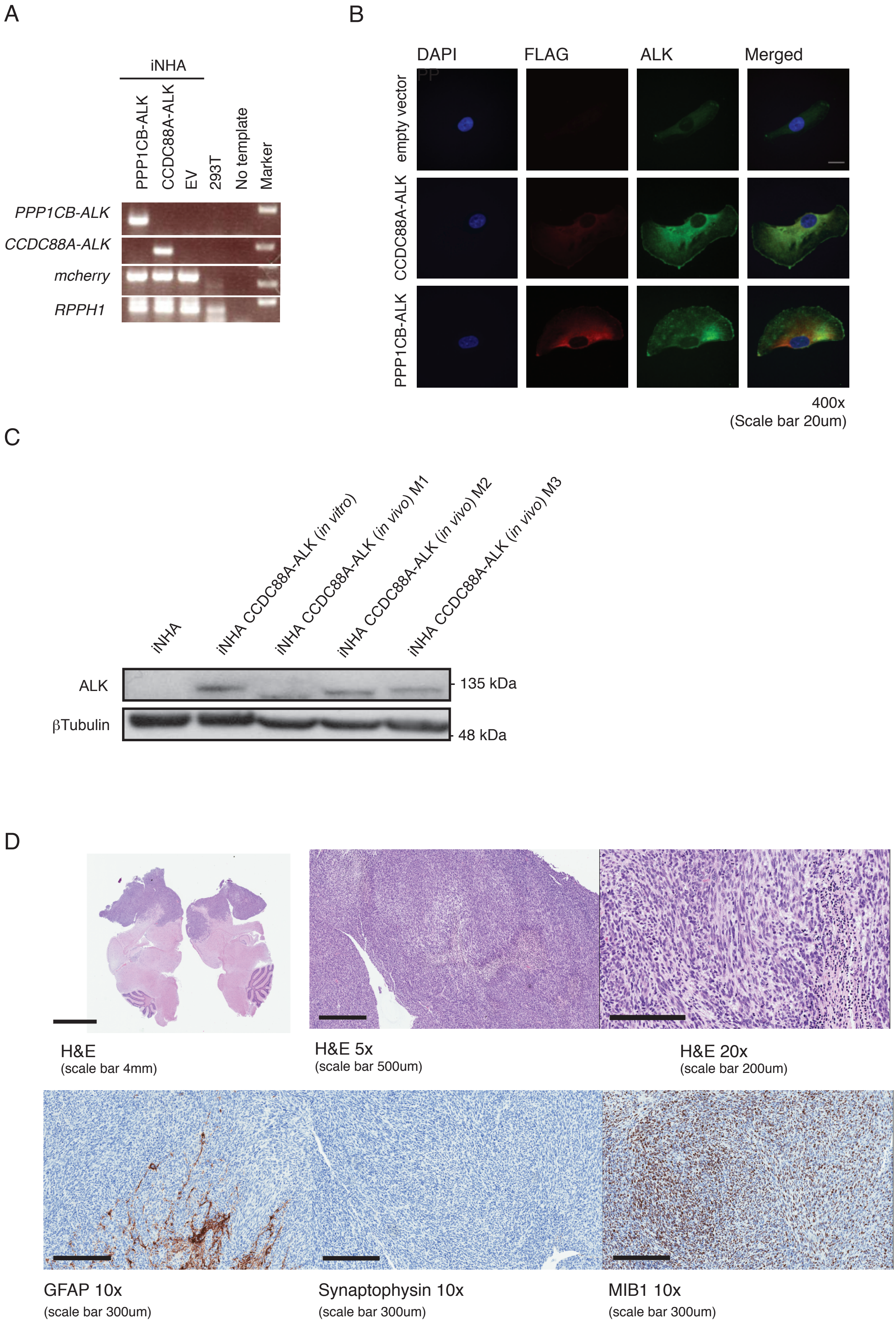

A
iNHA
PPP1CB-ALK
CCDC88A-ALK
EV
293T
No template
Marker
PPP1CB-ALK
CCDC88A-ALK
mcherry
RPPH1
B
DAPI
FLAG
ALK
Merged
empty vector
CCDC88A-ALK
PPP1CB-ALK
400x
(Scale bar 20um)
C
iNHA
iNHA CCDC88A-ALK (in vitro)
iNHA CCDC88A-ALK (in vivo) M1
iNHA CCDC88A-ALK (in vivo) M2
iNHA CCDC88A-ALK (in vivo) M3
ALK
βTubulin
135 kDa
48 kDa
D
H&E
(scale bar 4mm)
H&E 5x
(scale bar 500um)
H&E 20x
(scale bar 200um)
GFAP 10x
(scale bar 300um)
Synaptophysin 10x
(scale bar 300um)
MIB1 10x
(scale bar 300um)

Supplementary Figure 3. (A) PCRs showing the presence of mcherry, CCDC88A-ALK and PPP1CB-ALK in the respective stable iNHA cell lines; gDNA of 293T and no template controls included. RPPH1 used as house-keeping control gene. Marker band displayed is reflective of 250 basepairs. (B) Immunofluorescence showing ALK and FLAG expression in iNHA cells transduced with the empty vector, CCDC88A-ALK and PPP1CB-ALK. Images were taken at 400x, scale bar = 20 μM. (C) Western Blot showing overexpression of CCDC88A-ALK fusion protein. (D) Orthotopic xenografts of iNHAs overexpressing CCDC88A-ALK: Hemotoxylin and eosin (H&E) staining slides showing evidence of necrosis and multiple mitoses; GFAP focally positive, synaptophysin negative; proliferation index (MIB1) >50%. All images are taken at the stated magnification, scale bar = 100 μM, 200 μM for 4x unless otherwise specified in the figure.

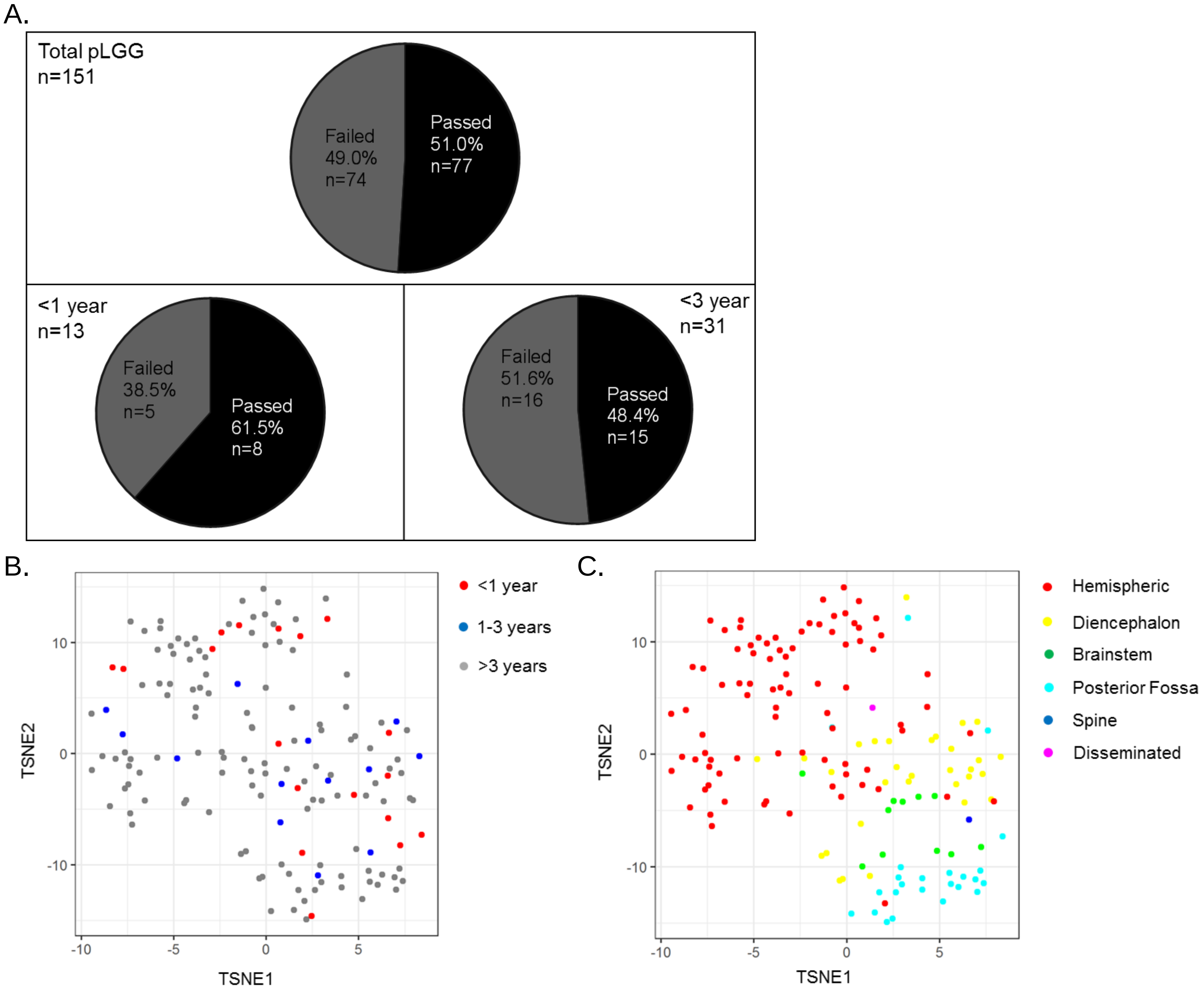
A.
Total pLGG
n=151
Failed
49.0%
n=74
Passed
51.0%
n=77
<1 year
n=13
Failed
38.5%
n=5
Passed
61.5%
n=8
<3 year
n=31
Failed
51.6%
n=16
Passed
48.4%
n=15
B.
<1 year
1-3 years
>3 years
TSNE1
TSNE2
C.
Hemispheric
Diencephalon
Brainstem
Posterior Fossa
Spine
Disseminated
TSNE1
TSNE2

Supplementary Figure 4. Methylation classification of infant glioma as compared to older pediatric low-grade glioma (LGG) tumors. (A) A graphical breakdown of the success rate at which infant and pediatric samples are confidently assigned a CNS classification based on Capper, *et. al*, 2018. (B) T-SNE plot showing that infant and pediatric tumors do not cluster with one another based on methylation, indicative that they are unique clinical entities. (C) T-SNE plot showing that infant and pediatric tumors cluster more readily based on the tumor's location rather than outcome.

T-SNE: t-Distributed Stochastic Neighbor Embedding

Characteristic		
Sex		
	Male	73
	Female	69
Histology		
	Low Grade	104
	High Grade	33
	Mixed	5
Location		
	Cerebral Hemispheres	71
	Diencephalon	52
	Posterior Fossa	8
	Brainstem	5
	Spinal Cord	6
Outcome		
	Alive	97
	Deceased	42
	Unknown	3
Progression		
	Progressed	84
	Stable	49
	Lost to Follow-Up	3
	Unknown	6
Extent of Surgery		
	None	3
	Biopsy	37
	Partial Resection	56
	Gross Total Resection	41
	Unknown	5
Radiation		
	Yes	18
	No	115
	Unknown	9
Chemotherapy		
	Yes	81
	No	53
	Unknown	8
Age at Diagnosis		
	Median (months)	6.8 (0.0-14.6)
	Mean (months)	6.7 ± 3.8
Progression-free Survival		
	Median (years)	1.2 (0.0-17.7)
	Mean (years)	3.3 ± 4.3
Overall Survival		
	Median (years)	4.6 (0.0-27.7)
	Mean (years)	6.8 ± 7.0

Supplementary Table 1. Clinical characteristics of the entire infant glioma cohort

		Univariate			Multivariate		
Variable		HR	95% C.I.	p Value	HR	95% C.I.	p Value
Overall							
	Sex (Female)	0.976	0.42-2.268	0.955	0.875	0.245-3.121	0.837
	Resection (Resected)	1.607	0.676-3.818	0.283	0.461	0.138-1.547	0.21
	BRAF Fusion (Positive)	0.826	0.305-2.234	0.707	0.746	0.179-3.111	0.688
	BRAFV600E (Positive)	1.18	0.414-3.369	0.757	0.55	0.109-2.789	0.471
	Chemotherapy (Yes)	1.157	0.981-3.514	0.796	0.253	0.06-1.069	0.062
	Radiation (Yes)	0.826	0.269-2.533	0.738	0.305	0.064-1.445	0.135
	Age (Infant)	12.839	2.957-55.753	0.001	27.084	3.882-188.982	0.001
Progression-Free							
	Sex (Female)	1.384	0.869-2.204	0.171	1.36	0.728-2.542	0.335
	Resection (Resected)	1.108	0.666-1.843	0.693	0.744	0.377-1.467	0.393
	BRAF Fusion (Positive)	0.934	0.554-1.574	0.798	1.21	0.498-2.937	0.674
	BRAFV600E (Positive)	1.775	1.031-3.058	0.039	1.299	0.543-3.106	0.557
	Chemotherapy (Yes)	4.506	2.15-9.441	<0.0001	4.305	1.706-10.865	0.002
	Radiation (Yes)	1.077	0.573-2.022	0.818	0.769	0.315-1.881	0.565
	Age (Infant)	2.857	1.794-4.551	<0.0001	2.386	1.154-4.934	0.019

Supplementary Table 2. Multivariate and univariate analysis of midline low-grade glioma

Genes	Exon 1	Exon 2	Event	Gene ID 1	Gene ID 2
PPP1CB-ALK	5	20	Fusion	NM_002709.2	NM_001353765.1
CCDC88A-ALK	12	20	Fusion	NM_001135597.1	NM_001353765.1
EML4-ALK	2	20	Fusion	NM_019063.4	NM_001353765.1
KTN1-ALK	41	20	Fusion	NM_001271014.1	NM_001353765.1
CEP85L-ROS1	12	35	Fusion	NM_001042475.2	NM_002944.2
GOPC-ROS1	8	35	Fusion	NM_001017408.2	NM_002944.2
TPM3-NTRK1	1	12	Fusion	NM_001043351.1	NM_001007792.1
NFASC-NTRK1	22	12	Fusion	NM_001005388.2	NM_001007792.1
BCAN-NTRK1	13	13	Fusion	NM_021948.4	NM_001007792.1
TPM3-NTRK1	1	9	Fusion	NM_001043351.1	NM_001007792.1
VCL-NTRK2	16	12	Fusion	NM_003373.3	NM_001007097.2
NTRK2-MID1	7	7	Fusion	NM_001007097.2	NM_000381.3
AGBL4-NTRK2	6	16	Fusion	NM_001323573.1	NM_001007097.2
SLMAP-NTRK2	1	16	Fusion	NM_001304420.2	NM_001007097.2
NAV1-NTRK2	1	11	Fusion	NM_001167738.1	NM_001007097.2
NTRK3-ETV6	13	5	Fusion	NM_001007156.2	NM_001987.4
ETV6-NTRK3	1	14	Fusion	NM_001987.4	NM_001007156.2
BTBD1-NTRK3	1	14	Fusion	NM_001011885.1	NM_001007156.2
TGF-MET	5	15	Fusion	NM_001007565.2	NM_000245.3
CLIP2-MET	12	15	Fusion	NM_003388.4	NM_000245.3
PTPRZ1-MET	1	1	Fusion	NM_001206838.1	NM_000245.3
PTPRZ1-MET	2	1	Fusion	NM_001206838.1	NM_000245.3
PTPRZ1-MET	8	1	Fusion	NM_001206838.1	NM_000245.3
FGFR2-CTNNA3	17	14	Fusion	XM_017015925.1	NM_001127384.2
ERC1-FGFR2	2	18	Fusion	NM_001301248.1	XM_017015925.1
FGFR2-INA	17	2	Fusion	XM_017015925.1	NM_032727.3
FGFR2-KIAA1598	17	7	Fusion	XM_017015925.1	N/A
FGFR2-ERC1	17	3	Fusion	XM_017015925.1	NM_001301248.1
QKI-RAF1	3	7	Fusion	NM_001301085.1	NM_001354689.1
MYB-QKI	9	5	Fusion	NM_001130172.1	NM_001354689.1
MYB-QKI	11	5	Fusion	NM_001130172.1	NM_001354689.1
MYB-QKI	15	5	Fusion	NM_001130172.1	NM_001354689.1
ALK WT	14	18	Reporter	NM_001353765.1	
ALK MUT	21	27	Reporter	NM_001353765.1	
ROS1 WT	27	30	Reporter	NM_002944.2	
ROS1 MUT	37	41	Reporter	NM_002944.2	
NTRK2 WT	4	10	Reporter	NM_001007097.2	
NTRK2 MUT	17	20	Reporter	NM_001007097.2	
ABCF1			Housekeeping	NM_001090.2	
ALAS1			Housekeeping	NM_000688.4	
CLTC			Housekeeping	NM_004859.2	
HPRT1			Housekeeping	NM_000194.1	

Supplementary Table 3. Fusion and reporter gene targets included on the NanoString 2 Panel used in this study. Exons, and transcript IDs are included
