# Supplementary material for: Alterations in ALK/ROS1/NTRK/MET drive a group of infantile hemispheric gliomas
Source: Nat Commun. 2019 Sep 25;10:4343. doi: 10.1038/s41467-019-12187-5 (PMC6761184; doi:10.1038/s41467-019-12187-5)
Supplement: Supplementary file 2 — Reporting Summary [file 41467_2019_12187_MOESM2_ESM.pdf]

nature research

Corresponding author(s): Cynthia Hawkins

Last updated by author(s): Jul 18, 2019

Reporting Summary

Nature Research wishes to improve the reproducibility of the work that we publish. This form provides structure for consistency and transparency in reporting. For further information on Nature Research policies, see Authors & Referees and the Editorial Policy Checklist.

Statistics

For all statistical analyses, confirm that the following items are present in the figure legend, table legend, main text, or Methods section.

n/a	Confirmed	
☐	☒	The exact sample size (*n*) for each experimental group/condition, given as a discrete number and unit of measurement
☐	☒	A statement on whether measurements were taken from distinct samples or whether the same sample was measured repeatedly
☐	☒	The statistical test(s) used AND whether they are one- or two-sided<br>*Only common tests should be described solely by name; describe more complex techniques in the Methods section.*
☐	☒	A description of all covariates tested
☒	☐	A description of any assumptions or corrections, such as tests of normality and adjustment for multiple comparisons
☐	☒	A full description of the statistical parameters including central tendency (e.g. means) or other basic estimates (e.g. regression coefficient) AND variation (e.g. standard deviation) or associated estimates of uncertainty (e.g. confidence intervals)
☐	☒	For null hypothesis testing, the test statistic (e.g. *F*, *t*, *r*) with confidence intervals, effect sizes, degrees of freedom and *P* value noted<br>*Give P values as exact values whenever suitable.*
☒	☐	For Bayesian analysis, information on the choice of priors and Markov chain Monte Carlo settings
☒	☐	For hierarchical and complex designs, identification of the appropriate level for tests and full reporting of outcomes
☒	☐	Estimates of effect sizes (e.g. Cohen's *d*, Pearson's *r*), indicating how they were calculated

Our web collection on statistics for biologists contains articles on many of the points above.

Software and code

Policy information about availability of computer code

Data collection	Data collection was completed as per each contributing institution's guidelines for clinical data acquisition. The data was provided to the referencing centre (Hospital for Sick Children) via a standard Microsoft Excel 2010 file where it was combined and securely stored.
Data analysis	Statistical analyses were performed using R version 3.5.0. and R Commander Version 2.4-4 with the plugins “Survival” (version 1.2-0), “KMggplot2” (version 0.2-5) and “Plot by Group” (version 0.1-0). No additional custom software was used.

For manuscripts utilizing custom algorithms or software that are central to the research but not yet described in published literature, software must be made available to editors/reviewers. We strongly encourage code deposition in a community repository (e.g. GitHub). See the Nature Research guidelines for submitting code & software for further information.

Data

Policy information about availability of data

All manuscripts must include a data availability statement. This statement should provide the following information, where applicable:
- Accession codes, unique identifiers, or web links for publicly available datasets
- A list of figures that have associated raw data
- A description of any restrictions on data availability

This manuscript contains the following as a data availability statement: The targeted and whole transcriptome sequencing data sets have been deposited in the European-Genome-phenome Archive under accession code EGAS00001003714. All other relevant data are available are available from the corresponding author upon request.

Field-specific reporting

Please select the one below that is the best fit for your research. If you are not sure, read the appropriate sections before making your selection.

☒ Life sciences ☐ Behavioural & social sciences ☐ Ecological, evolutionary & environmental sciences

For a reference copy of the document with all sections, see nature.com/documents/nr-reporting-summary-flat.pdf

Life sciences study design

All studies must disclose on these points even when the disclosure is negative.

Sample size	This manuscript aimed to molecularly characterize a critically under-represented demographic of infant gliomas. As such, we contacted collaborators in an attempt to accrue as many samples fitting this demographic as possible, and hence no "hard" sample size was set. As this collection represents the largest cohort of infant gliomas to our knowledge, we believe it to be of sufficient number to conclude on both the molecular and clinical trends observed. The number of samples reported on in each section of the manuscript is explicitly noted in the text.
Data exclusions	Data exclusion, as described in the text, was based on histological review revealing either no tumour present or a non-glioma diagnosis. A further set of samples were excluded due to insufficient biological material to garner experimental results. We also excluded patients diagnosed prior to 1985, as our past experience suggests that molecular characterization prior to this age to be exceedingly difficult.
Replication	All clinical findings are concluded on the largest cohort of infant gliomas assembled, to the best of our knowledge. We anticipate the replication of our clinical findings as additional samples are acquired from other institutions in the future. All in vitro experiments were completed in both technical and biological replicates to ensure validity. In vivo work was completed with randomized mouse groups and blinded end -point monitoring. All injections were completed in the same manner within the same 12 hour period.
Randomization	Randomization for the clinical aspects of this study was not completed, as the cohort as a whole was reported on. For the mice experiment, age and gender matched mice were randomly assigned to the experimental and control groups by a third party.
Blinding	Blinding of the researchers for the clinical portions of this study was not required. For mice work, endpoint monitoring was completed by technical services at our animal facility with no prior knowledge to which group each mouse was assigned to. Endpoints were defined by the same individual to ensure the absence of observer bias.

Reporting for specific materials, systems and methods

We require information from authors about some types of materials, experimental systems and methods used in many studies. Here, indicate whether each material, system or method listed is relevant to your study. If you are not sure if a list item applies to your research, read the appropriate section before selecting a response.

Materials & experimental systems

n/a	Involved in the study	
☐	☒	Antibodies
☐	☒	Eukaryotic cell lines
☒	☐	Palaeontology
☐	☒	Animals and other organisms
☒	☐	Human research participants
☐	☒	Clinical data

Methods

n/a	Involved in the study	
☒	☐	ChIP-seq
☒	☐	Flow cytometry
☒	☐	MRI-based neuroimaging

Antibodies

Antibodies used	1. Anti-ALK (D5F3(R)) XP(R) Rabbit mAB. Cell Signaling. Lot: 9 Cat: 3633<br>2. Anti-p44/42 MAPK (ERK1/2) Rabbit Ab, Cell Signaling, Lot: 27, Cat: 9102<br>3. Anti-P-p44/42 MAPK (T202/Y204) Rabbit Ab, Cell Signaling, Lot: 30, Cat: 9101<br>4. Anti-FLAG M2, Sigma, Lot: SLBS3530V, Cat: F1804<br>5. Tubulin (DM1A) Mouse mAb, Cell Signaling , Lot: 17, Cat: 2144<br>6. RTU anti-MIB-1 (mouse monoclonal primary antibody, ready-to-use, Dako Omnis), Cat: GA626<br>7. RTU anti-Synaptophysin (mouse monoclonal primary antibody, ready-to-use, Dako Omnis), Cat: GC202<br>8. RTU anti-GFAP (rabbit polyclonal primary antibody, ready-to-use, Dako Omnis), Cat: GA524
Validation	1. https://www.cellsignal.com/products/primary-antibodies/alk-d5f3-xp-rabbit-mab/3633<br>2. https://www.cellsignal.com/products/primary-antibodies/p44-42-mapk-erk1-2-antibody/9102<br>3. https://www.cellsignal.com/products/primary-antibodies/phospho-p44-42-mapk-erk1-2-thr202-tyr204-antibody/9101<br>4. https://www.sigmaaldrich.com/catalog/product/sigma/f1804?lang=en&region=CA<br>5. https://www.cellsignal.com/products/primary-antibodies/a-tubulin-antibody/2144<br>6. https://www.agilent.com/en/product/immunohistochemistry/antibodies-controls/primary-antibodies/ki-67-antigen-(dako-

omnis)-76239
7. https://www.agilent.com/en/product/immunohistochemistry/antibodies-controls/primary-antibodies/synaptophysin-(dako-omnis)-76264
8. https://www.agilent.com/en/product/immunohistochemistry/antibodies-controls/primary-antibodies/glial-fibrillary-acidic-protein-(dako-omnis)-76214

Eukaryotic cell lines

Policy information about cell lines

Cell line source(s)	Immortalized normal human astrocytes were a gift from a collaborator that have since been cultured in our lab Reference of collaborator: Sonoda, Y. et al. Formation of intracranial tumors by genetically modified human astrocytes defines four pathways critical in the development of human anaplastic astrocytoma. Cancer Res 61, 4956-60 (2001).
Authentication	Cell lines were not authenticated.
Mycoplasma contamination	Cells lines were not tested for mycoplasma, but are routinely treated with "MycoZap" (Lonza, Germany). Cat #: VZA-2031. Lot: 923D1126 to ensure the absence of mycoplama contamination.
Commonly misidentified lines (See ICLAC register)	No commonly misidentified cell lines were used in this study.

Animals and other organisms

Policy information about studies involving animals; ARRIVE guidelines recommended for reporting animal research

Laboratory animals	Nod/Scid/Gamma mice were injected at 8 weeks of age. All male mice were used.
Wild animals	This study did not involve wild animals.
Field-collected samples	This study did not involve field-collected samples.
Ethics oversight	The study was approved by the Animal Care Committee at The Centre for Phenogenomics (Toronto, ON, CAN), the animal facility partner at the Hospital for Sick Children (Toronto, ON, CAN)

Note that full information on the approval of the study protocol must also be provided in the manuscript.

Clinical data

Policy information about clinical studies
All manuscripts should comply with the ICMJE guidelines for publication of clinical research and a completed CONSORT checklist must be included with all submissions.

Clinical trial registration	This study did not include a clinical trial.
Study protocol	This study was not include a clinical trial
Data collection	Clinical data collected was provided by the treating oncologist at each respective institution. Histological grade and post-operative information was provided by the providing institutes pathologist and neurosurgeon/oncologist, respectively.
Outcomes	Outcome data was provided by the contributing institution. Progression events were defined as changes in clinical behavior and/or tumor that resulted in a change of clinical management as determined by the treating physician.
